# Inhibition of the Tcf/beta-catenin complex increases apoptosis and impairs adrenocortical tumor cell proliferation and adrenal steroidogenesis

**DOI:** 10.18632/oncotarget.5513

**Published:** 2015-10-16

**Authors:** Letícia F. Leal, Ana Carolina Bueno, Débora C. Gomes, Rafael Abduch, Margaret de Castro, Sonir R. Antonini

**Affiliations:** ^1^ Department of Pediatrics, Ribeirao Preto Medical School, University of Sao Paulo, Ribeirao Preto, Sao Paulo, Brazil; ^2^ Department of Pediatrics, School of Medicine, Federal University of Uberlandia, Uberlândia, Minas Gerais, Brazil; ^3^ Department of Internal Medicine, Ribeirao Preto Medical School, University of Sao Paulo, Ribeirao Preto, Sao Paulo, Brazil

**Keywords:** adrenocortical cancer, beta-catenin, steroidogenesis, apoptosis, targeted therapy

## Abstract

**Background:**

To date, there is no effective therapy for patients with advanced/metastatic adrenocortical cancer (ACC). The activation of the Wnt/beta-catenin signaling is frequent in ACC and this pathway is a promising therapeutic target.

**Aim:**

To investigate the effects of the inhibition of the Wnt/beta-catenin in ACC cells.

**Methods:**

Adrenal (NCI-H295 and Y1) and non-adrenal (HeLa) cell lines were treated with PNU-74654 (5–200 μM) for 24–96 h to assess cell viability (MTS-based assay), apoptosis (Annexin V), expression/localization of beta-catenin (qPCR, immunofluorescence, immunocytochemistry and western blot), expression of beta-catenin target genes (qPCR and western blot), and adrenal steroidogenesis (radioimmunoassay, qPCR and western blot).

**Results:**

In NCI-H295 cells, PNU-74654 significantly decreased cell proliferation 96 h after treatment, increased early and late apoptosis, decreased nuclear beta-catenin accumulation, impaired *CTNNB1*/beta-catenin expression and increased beta-catenin target genes 48 h after treatment. No effects were observed on HeLa cells. In NCI-H295 cells, PNU-74654 decreased cortisol, testosterone and androstenedione secretion 24 and 48 h after treatment. Additionally, in NCI-H295 cells, PNU-74654 decreased *SF1* and *CYP21A2* mRNA expression as well as the protein levels of STAR and aldosterone synthase 48 h after treatment. In Y1 cells, PNU-74654 impaired corticosterone secretion 24 h after treatment but did not decrease cell viability.

**Conclusions:**

Blocking the Tcf/beta-catenin complex inhibits the Wnt/beta-catenin signaling in adrenocortical tumor cells triggering increased apoptosis, decreased cell viability and impairment of adrenal steroidogenesis. These promising findings pave the way for further experiments inhibiting the Wnt/beta-catenin pathway in pre-clinical models of ACC. The inhibition of this pathway may become a promising adjuvant therapy for patients with ACC.

## INTRODUCTION

The management of patients with adrenocortical carcinomas (ACCs) remains a challenge and patients with invasive, metastatic or recurrent disease have a poor prognosis [1, 2]. Although significant progress has been achieved in recent years both in basic and clinical research, adjuvant therapeutic options for patients with ACC remain very limited [3]. Mitotane (M), the first line adjuvant treatment, is highly toxic and its combination with etoposide, doxorubicin, and cisplatin (EDP) is generally ineffective and almost all patients will experience disease progression [4]. New molecular targeted therapies have been successfully developed for various types of cancers but not for ACCs. Thus, better treatments are urgently necessary.

Several genetic abnormalities have been found in these tumors, the most prominent being IGF2 overexpression [5–7] and mutations in *TP53* and *CTNNB1* (the beta-catenin gene) in both adult and pediatric adrenocortical tumors (ACTs) [8–10]. Transcriptome studies have shown that ACCs are clustered within different sets of poor prognosis for adult ACC patients according to *TP53* or *CTNNB1* abnormalities [10]. Accordingly, overexpression of beta-catenin in ACCs has been correlated with a worse prognosis [11]. Exon 3 *CTNNB1* mutations have been found in 15–36% and 6% of adult and pediatric ACTs, respectively [8, 9, 12–15]. We previously showed that activation of both canonical and non-canonical Wnt signaling pathways are common in ACTs with or without *CTNNB1* mutations [8, 9]. The hypothesis that the Wnt pathway can be activated through other mechanisms than *CTNNB1* mutations has been recently reinforced. A large-scale high-resolution analysis study showed that variations in *ZNRF3,* which is a Wnt/beta-catenin pathway inhibitor, were the most common genetic defect found in a large number of ACC samples. ACCs presenting *ZNRF3* variants showed transcriptional activation of beta-catenin target genes [16]. Thus, activation of the Wnt/beta-catenin pathway triggered by *CTNNB1* and *ZNRF3* mutations or down regulation of Wnt/beta-catenin inhibitors are important for ACC pathogenesis. Therefore, inhibition of the Wnt/beta-catenin signaling is a rational option and may become a promising approach.

*CTNNB1* mutations found in ACCs are located at residues involved in phosphorylation, which are essential sites for beta-catenin degradation by ubiquitin/proteasome signaling. Therefore, mutations in these sites lead to beta-catenin accumulation in the nucleus, where it binds with the T cell factor (Tcf) and enhances its transcriptional activity [15]. The NCI-H295 cell line is an immortalized adrenocortical-secreting carcinoma lineage derived from an adult patient [17]. Remarkably, this cell line harbors the *CTNNB1* p.S45P mutation, thus representing a good *in vitro* model of ACC showing Wnt/beta-catenin pathway activation [14, 15]. High-throughput screening identified small molecules that antagonize the Tcf/beta-catenin complex and inhibit the growth of tumor cell lines [18]. Among Tcf/beta-catenin antagonists, PKF115-584 has been reported to inhibit proliferation of the NCI-H295R cell line and the expression of the beta-catenin target genes cyclin D1 and c-Myc [19].

**Figure 1:**
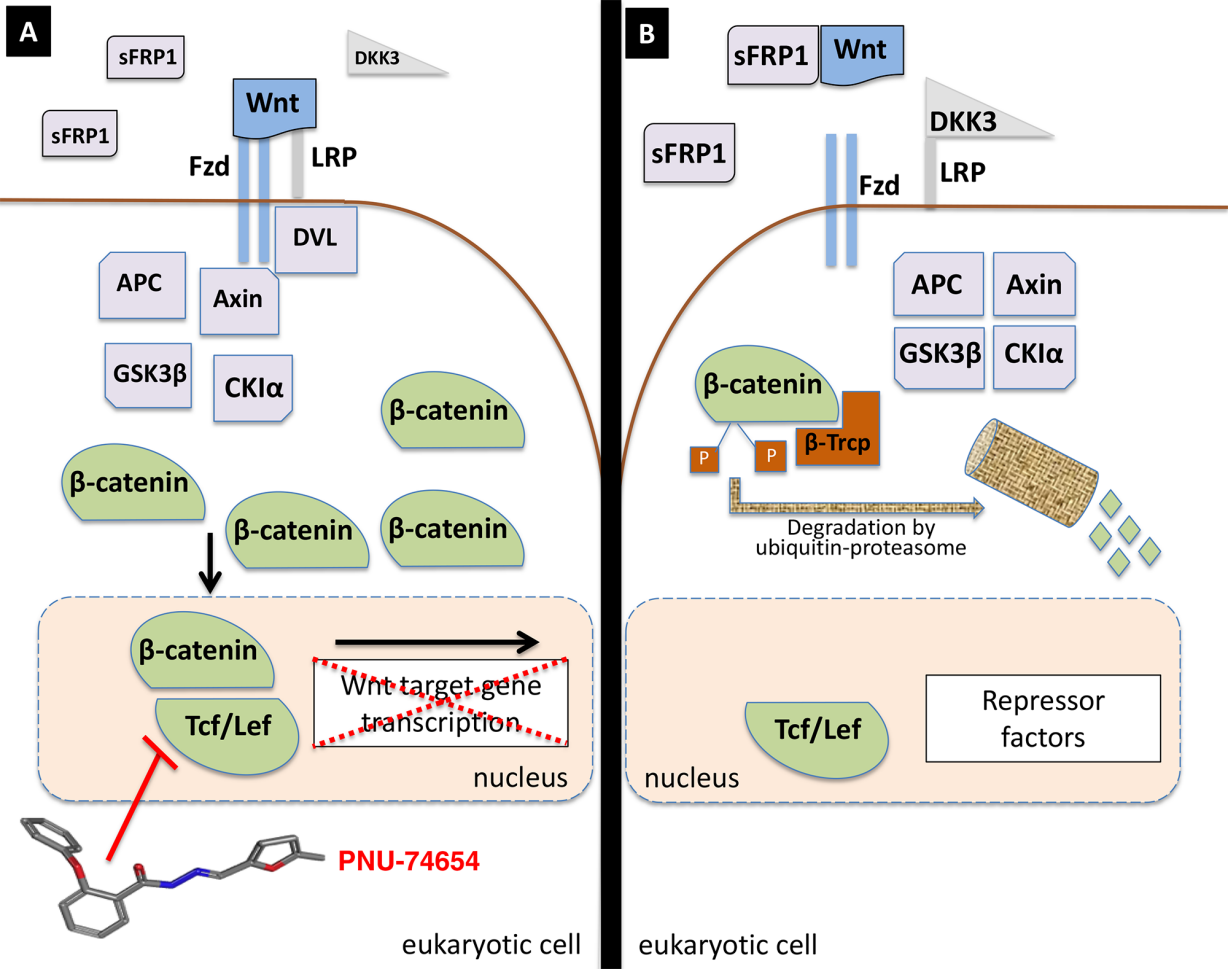
Wnt pathway signaling and PNU-74654 effect on the Tcf/beta-catenin complex **A.** When Wnt signaling is activated, the Wnt ligand binds to the Frizzled (Fzd) receptor and LRP5/6 (LRP) co-receptor and stimulates LRP5/6 phosphorylation with the help of Dishevelled (DVL). Phosphorylated LRP recruits Axin to the membrane and disrupts the beta-catenin degradation complex. Beta-catenin accumulates in the cytoplasm and enters into the nucleus, where it binds to Tcf/Lef and co-activators triggering Wnt target gene transcription. PNU-74654, a drug-like compound, disrupts the beta-catenin/Tcf complex and arrests Wnt target gene transcription. **B.** When Wnt signaling is not activated (either by Wnt ligand sequestration by sFRPs and/or LRP5/6 inhibition by DKK3), cytoplasmic beta-catenin is phosphorylated by the beta-catenin degradation complex consisting of APC, Axin, GSK3beta and CKIα. Phosphorylated beta-catenin is then recognized by beta-Trcp and is degraded via the ubiquitin-proteasome pathway.

The PNU-74654 (PNU) compound is a non-FDA-approved drug which prevents that Tcf from binding to beta-catenin, acting as a Wnt/beta-catenin antagonist (Figure 1). This small molecule was found by virtual screening and confirmed by biophysical screening to interfere with protein-protein interactions [20]. Beta-catenin tightly binds to Tcf through a hot spot site. By binding to the same site, PNU can compete with Tcf. A luciferase activity assay for Tcf transactivation showed specific inhibition in the presence of PNU, confirming that this drug-like compound is an effective Wnt pathway antagonist [20].

Taken together, these data suggest that the Wnt/beta-catenin pathway might be a potential targeted therapy for patients with ACC. Tcf/beta-catenin antagonism may be useful to treat patients with ACC exhibiting increased Wnt/beta-catenin signaling. In the present study, we assessed the *in vitro* effect of PNU on adrenocortical tumor cells. Our results showed that inhibition of the Wnt/beta-catenin signaling resulted in a significantly decreased cell viability, increased apoptosis and impaired steroidogenesis.

## RESULTS

### Authentication and sequencing analysis of exon 3 *CTNNB1* and *TP53* coding regions in cell lines

**Figure 2:**
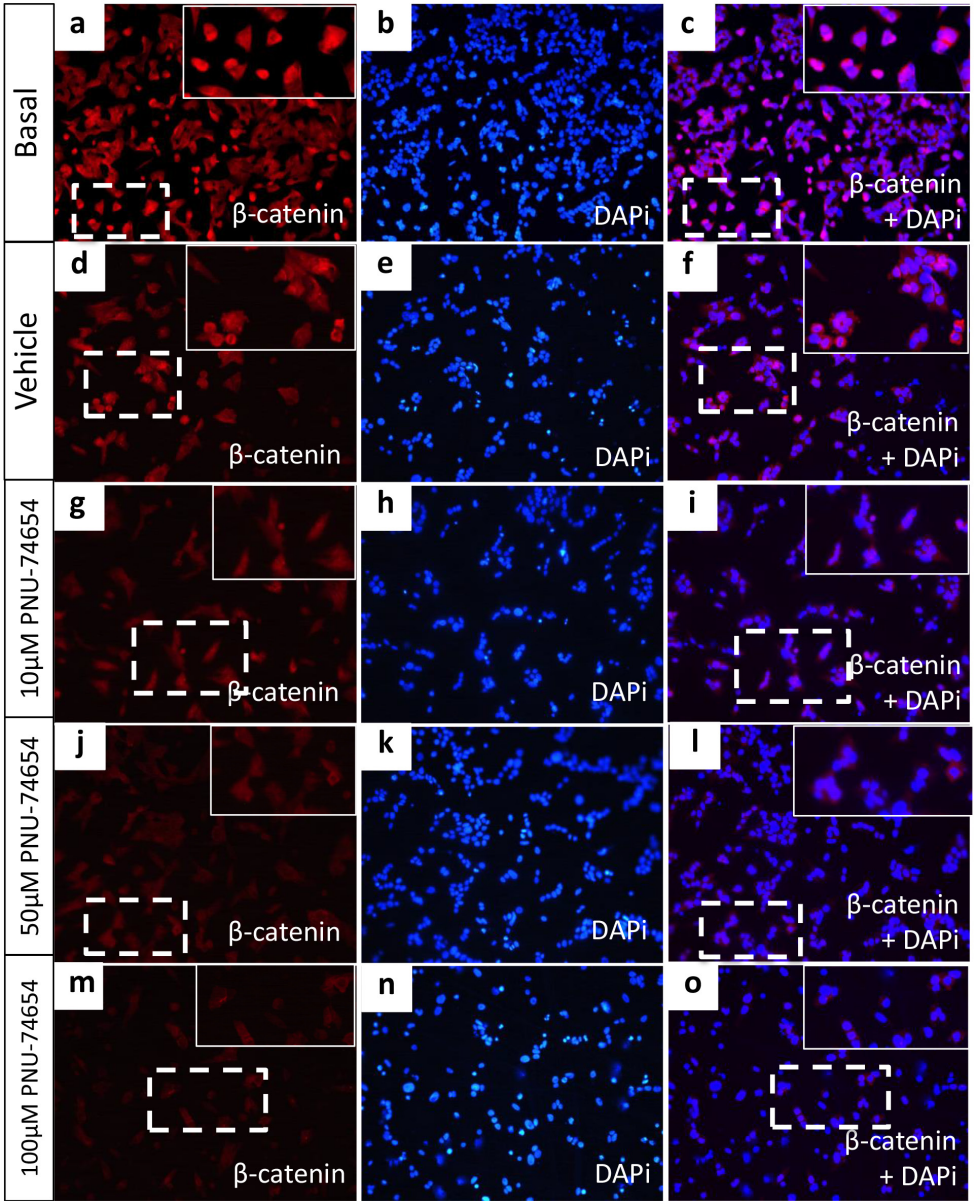
Beta-catenin immunofluorescence in the NCI-H295 cell line **(a-f)** NCI-H295 cells displayed nuclear and cytoplasmic beta-catenin expression under basal conditions and 48 h after treatment with vehicle (DMSO). **(g–i)** NCI-H295 cells displayed decreased nuclear and cytoplasmic beta-catenin expression 48 h after treatment with 10 μM PNU-74654, **(j–l)**, reduced cytoplasmic and absent nuclear beta-catenin expression after 50 μM PNU-74654 and **(m–o)** and very low cytoplasmic beta-catenin nuclear expression after 100 μM PNU-74654 treatment. Blue = nuclear staining (DAPi 1:25000; Cell Signaling Technology); red = beta-catenin (anti-beta-catenin 1:2000; BD Biosciences); pink = merged.

NCI-H295 and HeLa authenticity was confirmed by STR profiling (Supplementary Table 1). In our own stock of NCI-H295 cells, we confirmed the presence of the pathogenic p.S45P *CTNNB1* variation. Immunofluorescence analysis showed that, under basal conditions, beta-catenin is highly expressed in the cytoplasm and nucleus of NCI-H295 cells (Figure 2a–2c). This finding confirms that the Wnt/beta-catenin pathway is activated in NCI-H295 cells. In addition, we also detected the presence of the p.P278L *TP53* variation in this cell line.

HeLa, a cervix carcinoma cell lineage, was employed as a non-adrenal cell line that did not carry the exon 3 *CTNNB1* mutation but which also showed the p.P72R *TP53* variation as well as a second intronic *TP53* variation (c.97-30C > A/g.11298). Additionally, we identified an intronic *TP53* variation at position c.74 + 38 (C > G rs1642785) in HeLa cells. These results are summarized in Supplementary Table 2.

### Inhibition of the Tcf/beta-catenin complex induced by PNU impairs cell viability in human adrenocortical cell lines but not in mouse adrenal cell lines and human non-adrenal cell lines and prevents cell proliferation by Forskolin stimulation (Figures 3 and 4)

**Figure 3:**
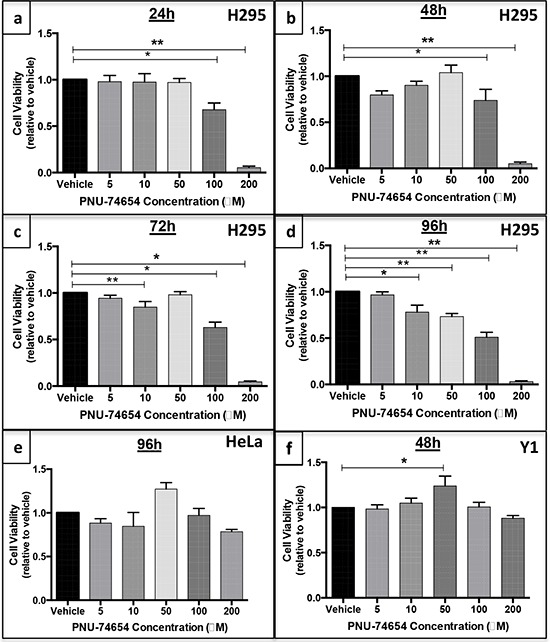
PNU-74654 treatment reduces cell viability in human adrenocortical cell lines but not in a mouse adrenal cell line or human non-adrenal cell line NCI-H295 cell viability 24 **a.** 48 **b.** 72 **c.** and 96 **d.** hours after treatment with 5, 10, 50, 100 and 200 μM PNU-74654. HeLa cell viability 96 **e.** hours after treatment with 5, 10, 50, 100 and 200 μM PNU-74654. Y1 cell viability 48 h **f.** after treatment with 5, 10, 50, 100 and 200 μM PNU-74654. Cell viability was calculated by relative absorbance normalized by vehicle (DMSO). Values are reported as mean ± SEM. Statistics: ANOVA (a) *p = 0.0012; **p < 0.0001 (b) *p = 0.02; **p < 0.0001; (c) *p < 0.0001; **p = 0.03 (d) *p = 0.001, **p < 0.0001; (e) *p* = 0.05; (f) *p = 0.04.

PNU impaired NCI-H295 cell viability. NCI-H295 cell viability was decreased 24 and 48 h after treatment with 100 and 200 μM PNU (Figure 3a–3b) and 72 h after treatment with 10, 100 and 200 μM PNU (Figure 3c). Lower doses of PNU were able to decrease cell viability 96 h after treatment: 22, 27, 50 and 97% with 10, 50, 100 and 200 μM, respectively (ANOVA: *p* < 0.0001; Figure 3d).

PNU did not decrease HeLa cell viability 96 h after treatment compared to vehicle (*p* = 0.05; Figure 3e). Interestingly, PNU showed a weak effect 48 h after treatment, but this effect was observed only at a concentration of 200 μM (ANOVA: *p* = 0.01; Supplementary Figure 1).

PNU did not decrease Y1 cell viability 48 h after treatment (Figure 3f). We chose to evaluate these cells up to 48 h and not at 96 h because they grow much faster than NCI-H295 cells under culture.

**Figure 4:**
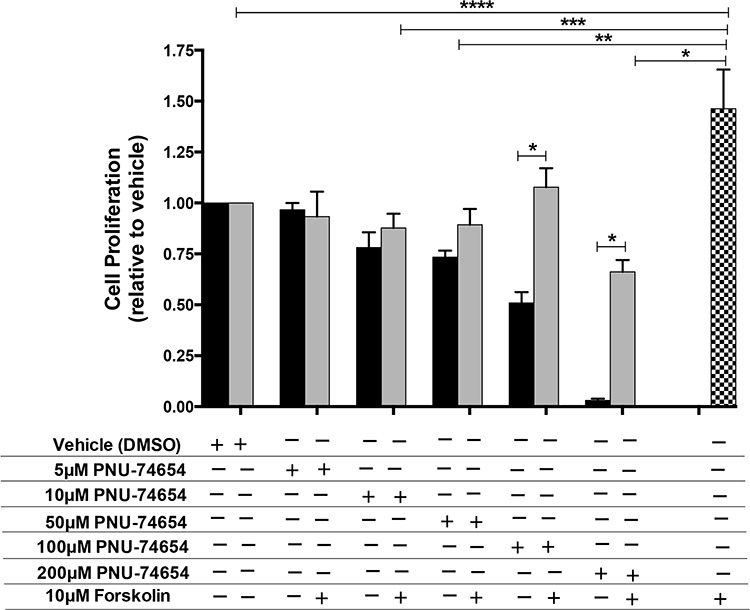
PNU-74654 treatment prevents Forskolin-induced cell proliferation in NCI-H295 cells Forskolin stimulation increased cell proliferation compared with vehicle and compared with PNU treatment alone and under Forskolin stimulation (*p = 0.0007; **p = 0.03; ***p = 0.02; ****p = 0.0008). PNU treatment under Forskolin stimulation compared with PNU 100 and 200 μM (*p < 0.0001). Black bars: PNU treatment alone; Gray bars: PNU treatment under Forskolin stimulation; Black/white bar: Forskolin stimulation alone. Values are reported as mean ± SEM relative to vehicle of 3 independent experiments.

As expected, 10 μM Forskolin increased cell proliferation by approximately 50% compared with vehicle (*p* = 0.0008; Figure 4). In turn, association of 5, 10 and 50 μM PNU with Forskolin prevented Forskolin-induced cell proliferation 96 h after treatment (Figure 4).

### Inhibition of the Tcf/beta-catenin complex induced by PNU increased NCI-H295 cell apoptosis (Figure 5)

**Figure 5:**
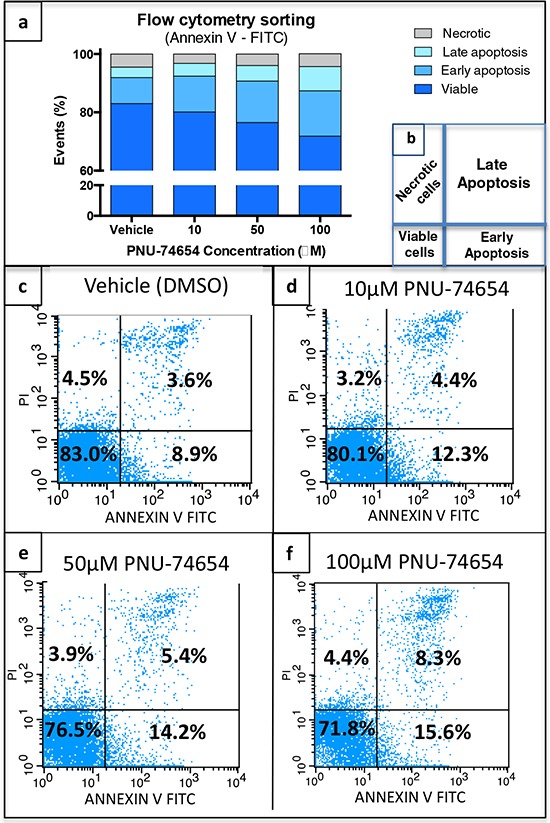
Tcf/Beta-catenin complex inhibition increases apoptotic cells **a, c–f.** PNU-75654 induced an increase of late and early apoptotic cells 48 h after treatment with 10, 50 and 100 μM. **b.** Schematic figure displaying flow cytometry squares. (c–f) Representative figures of flow cytometry sorting of NCI-H295 cells 48 h after treatment with (c) vehicle, (d) 10, (e) 50, and (f) 100 μM PNU-74654. (a, c–f) Values are reported as mean percentage (%) of 3 independent experiments.

PNU treatment at the concentrations 10, 50 and 100 μM resulted in a dose-dependent increase (19%, 51% and 80%, respectively) of late apoptotic cells 48 h after treatment (Figure 5a and 5c–5f). PNU treatment at the concentrations of 10, 50 and 100 μM also resulted in an increase (18%, 60% and 31%, respectively) of early apoptotic cells 48 h after treatment (Figure 5a and 5d–5g). The percentage of necrotic cells was decreased after PNU treatment at the concentrations of 10, 50 and 100 μM (−10%, −15% and −4%, respectively; Figure 5a and 5c–5f). The reduction of NCI-H295 cell viability was confirmed 48 h after treatment with 50 and 100 μM by flow cytometry sorting (*p* = 0.04 and *p* = 0.01, respectively; Figure 4a). Schematic flow cytometry squares are characterized in Figure 4b.

### Inhibition of the Tcf/beta-catenin complex induced by PNU decreases *CTNNB1* mRNA expression and nuclear beta-catenin accumulation but not TCF expression and changes beta-catenin target genes expression in NCI-H295 cells (Figures 2, 6 and 7)

**Figure 6:**
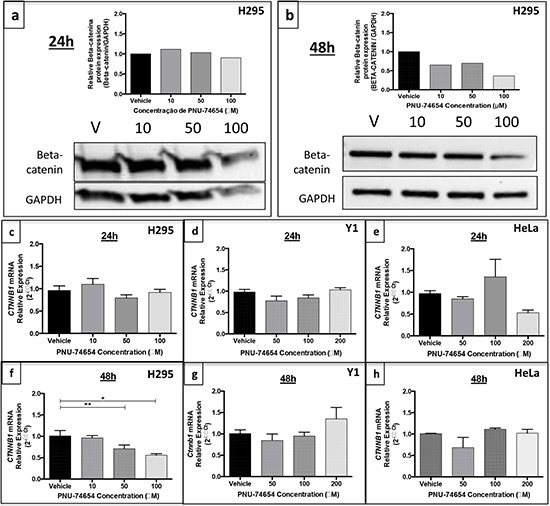
Beta-catenin expression in the NCI-H295 human adrenal, Y1 mouse adrenal and HeLa non-adrenal cell lines after PNU-74654 treatment Beta-catenin protein expression after 24 **a.** and 48 h **b.** after treatment with 10, 50 and 100 μM PNU-74654 (beta-catenin antibody: 92 kDa; 1:1000; BD Biosciences/GAPDH antibody: 37kDa; 1:1000; Santa Cruz). *CTNNB1*/beta-catenin mRNA expression in NCI-H295 cells 24 **c.** and 48 h **f.** after treatment with 10, 50 and 100 μM PNU-74654. (f) *p = 0.003, **p = 0.04. Ctnnb1 mRNA expression in Y1 cells after 24 **d.** and 48 h **g.** after treatment with 50, 100 and 200 μM PNU-74654. *CTNNB1* mRNA expression in HeLa cells after 24 **e.** and 48 h **h.** after treatment with 50, 100 and 200 μM PNU-74654. V: vehicle. Values are reported as mean ± SEM.

Immunofluorescence analysis showed a marked decrease of nuclear and cytoplasmic beta-catenin expression 48 h after treatment with 10, 50 and 100 μM PNU compared with vehicle (Figure 2d–2o). Similarly, beta-catenin/*CTNNB1* mRNA expression was decreased 48 h after PNU treatment with 50 (*p* = 0.04, fold change = −1.5; Figure 6b) and 100 μM (*p* = 0.0034, fold change = −1.8; Figure 6b) as also was beta-catenin protein expression (Figure 6a and 6b). *Ctnnb1/CTNNB1* mRNA expression was not decreased by PNU treatment in Y1 or HeLa cells (Figure 6d-g and 6e-h, respectively).

Immunostaining analysis showed a membranous and cytoplasmic pattern of beta-catenin accumulation in Y1 cells under basal conditions (Supplementary Figure 2). This result supports the idea that the Y1 cell line does not present Wnt/beta-catenin activation.

**Figure 7:**
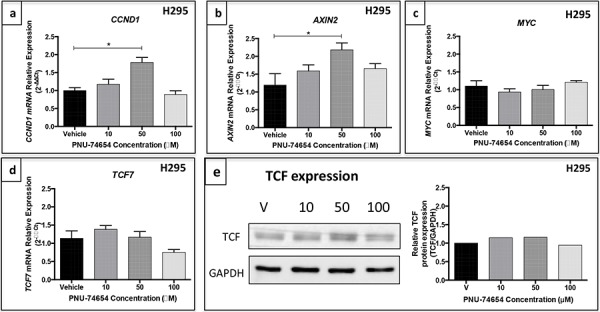
Gene expression of beta-catenin target genes in the NCI-H295 cell line after PNU-74654 treatment **a.** *CCND1* mRNA expression was increased in NCI-H295 cells 48 h after treatment with 50 μM PNU-74654. *p = 0.005. **b.** *AXIN2* mRNA expression was increased in NCI-H295 cells 48 h after treatment with 50 μM PNU-74654. *p = 0.01. **c.** *MYC* mRNA expression was not decreased in NCI-H295 cells 48 h after treatment with 100 μM PNU-74654 (*p* = 0.17). *TCF7* mRNA expression **d.** and TCF protein expression **e.** were not decreased in NCI-H295 cells 48 h after treatment with PNU-74654 (*p* = 0.02). V: vehicle. Values are reported as mean ± SEM.

Regarding beta-catenin target genes, 50 μM PNU increased the mRNA expression of *CCND1* (*p* = 0.005, fold change: 1.86; Figure 7a) and *AXIN2* (*p* = 0.01, fold change = 2; Figure 7b) 48 h after treatment. On the other hand, *MYC* mRNA expression (Figure 7c) and *TCF* mRNA and protein expression (Figure 7d–7e) did not change after PNU treatment.

### Inhibition of the Tcf/beta-catenin complex induced by PNU impairs adrenal steroidogenesis in NCI-H295 and Y1 cells (Figures 8 and 9)

**Figure 8:**
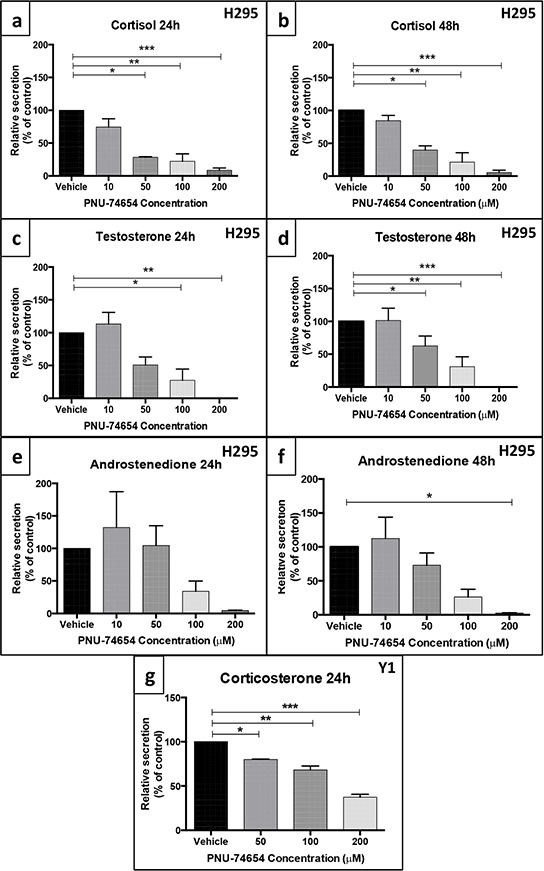
PNU-74654 treatment impaired adrenal steroidogenesis in the NCI-H295 and Y-1 cell lines In NCI-H295 cells, treatment with 50, 100 and 200 μM PNU-74654 reduced cortisol secretion at 24 **a.** and 48 h **b.** after treatment. (a) *p = 0.0006; **p = 0.0003; ***p = 0.0002; (b) *p = 0.002; **p = 0.0003; ***p = 0.0002. In NCI-H295 cells, treatment with 50, 100 and 200 μM PNU-74654 reduced testosterone secretion 24 **c.** and 48 h **d.** after treatment. (c) *p = 0.01; **p = 0.003; (d) *p = 0.02; **p = 0.0005; ***p < 0.0001. In NCI-H295 cells, PNU-74654 treatment did not decrease androstenedione secretion 24 h after treatment **e.** but reduced androstenedione secretion at 200 μM 48 h after treatment **f.** *p = 0.02. **g.** In Y-1 cells, treatment with 50, 100 and 200 μM PNU-74654 reduced corticosterone secretion 24 h after treatment (*p = 0.02, **p = 0.003, ***p = 0.0002). Values are reported as mean ± SEM percentage relative to vehicle of at least 3 independent experiments.

Under basal conditions, NCI-H295 cells retain the ability to secrete adrenal steroids [21]. Overall, PNU treatment significantly impaired adrenal steroidogenesis by decreasing steroid production and the mRNA expression of steroidogenesis-related genes in NCI-H295 cells. PNU decreased cortisol production by 72 and 78% (50 and 100 μM, respectively; ANOVA: *p* = 0.0002; Figure 8a) 24 h after treatment and by 60 and 78% (50 and 100 μM, respectively; ANOVA: *p* = 0.0001; Figure 8b) 48 h after treatment. PNU decreased testosterone production by 50 and 73% (50 and 100 μM, respectively; ANOVA: *p* = 0.0012; Figure 8c) 24 h after treatment and by 37 and 69% (50 and 100 μM, respectively; ANOVA: *p* < 0.0001; Figure 8d) 48 h after treatment. PNU decreased androstenedione production by 97.5% at 200 μM (ANOVA: *p* = 0.02; Figure 8e and 8f) 48 h after treatment.

PNU treatment also impaired steroid production in adrenal mouse cells. Corticosterone secretion was reduced by 20, 32 and 63% 24 h after treatment with 50, 100 and 200 μM PNU, respectively (ANOVA: *p* = 0.0004; Figure 8g).

**Figure 9:**
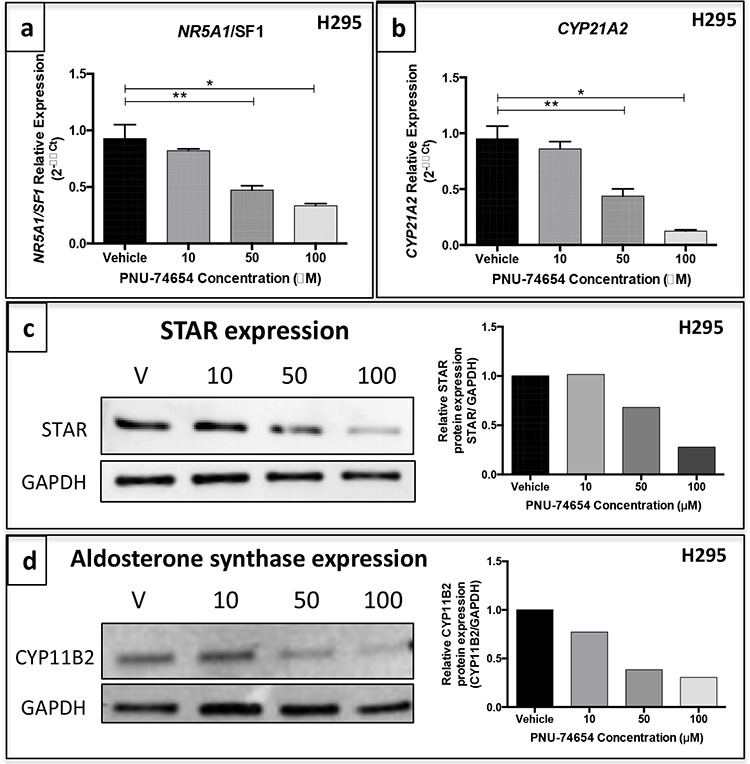
PNU-74654 treatment decreased the expression of steroidogenesis key regulatory enzymes in the NCI-H295 cell line *NR5A1*/SF1 **a.** and *CYP21A2* **b.** mRNA expression was decreased in NCI-H295 cells 48 h after treatment with 50 and 100 μM PNU-74654. *p < 0.0001; **p < 0.001. Values are reported as mean ± SEM. **c.** STAR protein expression in NCI-H295 cells 48 h after treatment with 10, 50 and 100 μM PNU-74654 showed by western blot. **d.** Aldosterone synthase protein expression in NCI-H295 cells 48 h after treatment with 10, 50 and 100 μM PNU-74654 shown by western blot. Values are plotted graphically. GAPDH was used as endogenous/loading control. V: vehicle.

PNU at 50 and 100 μM decreased *NR5A1/SF1* (52 and 66%, respectively; ANOVA: *p* < 0.0001; Figure 9a) and *CYP21A2* mRNA expression (63 and 88%, respectively ANOVA: *p* < 0.0001; Figure 9b) 48 h after treatment. PNU at 50 and 100 μM also decreased STAR relative protein expression 48 h after treatment (32 and 77%, respectively; Figure 9c). Additionally, PNU markedly decreased the relative protein expression of aldosterone synthase 48 h after treatment with 10, 50 and 100 μM PNU (23%, 62% and 70%, respectively; Figure 9d).

## DISCUSSION

In the present study we demonstrated the anti-proliferative, pro-apoptotic and anti-steroidogenic effects of the Wnt/beta-catenin pathway inhibition in ACC cell lines. In addition, we provided evidence that PNU may directly impair adrenal steroid secretion. To date, there is no effective adjuvant therapy for patients with advanced or metastatic ACCs. Very recently, a phase II clinical trial involving the suppression of the IGF system was tested in ACC patients but unfortunately the results were rather disappointing [22]. Hence, new approaches based on molecular targeted therapies are suitable.

Abnormal activation of the Wnt/beta-catenin pathway plays an important role in adrenocortical tumorigenesis [8, 10, 12, 15, 23]. Actually, Wnt/beta-catenin-related abnormalities appear to be the most common molecular signature of these tumors [8–10, 12, 14–16, 24]. In addition, beta-catenin constitutive activation in mice results in adrenal glands with malignant characteristics, such as uncontrolled neovascularization and loco-regional metastatic invasion [25]. In agreement, a previous study confirmed overexpression of Wnt target genes in ACTs harboring beta-catenin mutations as well as in the adrenal cell lineage NCI-H295. In that study, 24 h treatment with Wnt inhibitors, 10 μM PKF115-584 and 100 μM PNU, resulted in decreased beta-catenin target gene transcription [24]. However, the impact of these compounds on apoptosis, cell viability and steroidogenesis has not been assessed. Our study investigated in depth the effects of PNU on cell viability over a longer period of time (96 h). Moreover, we also determined its effects on apoptosis and adrenal steroidogenesis.

A previous study had shown that PKF115-584, currently called Calphostin C, dose-dependently inhibited beta-catenin-dependent transcription and H295R proliferation, and induced entry of cells into the S phase [19]. However the topological polar surface of PKF115-584 is 194 square angstrom (Å^2^), higher than recommended for good absorption (less than 140 Å^2^), characterizing this compound as poorly absorbed by cells [26]. On the other hand, the topological surface of PNU-74654 is 63.8 Å^2^ causing this compound to be very well absorbed by cells for a better effect on Wnt signaling inhibition (National Center for Biotechnology Information. PubChem Compound Database; CID = 60119583, https://pubchem.ncbi.nlm.nih.gov/compound/60119583 (accessed Aug. 12, 2015).) [27]. In our study, PNU was able to decrease cell viability since 24 h after treatment at higher but apparently non-cytotoxic concentrations (100 μM). After a longer time of treatment (72 h and 96 h) this effect was achieved even with a lower dose (10 μM). Additionally, we analyzed the impact of this drug under conditions of stimulation with Forskolin, which has a potent effect on adrenal cell proliferation. Forskolin alone increased cell proliferation by approximately 50%. In contrast, PNU prevented the cell proliferation stimulated by Forskolin. The effects of PNU appear to be partially specific for cells carrying Wnt/beta-catenin activation. Our results showed that PNU does not decrease cell proliferation in cells that do not carry exon 3 beta-catenin mutations, such as HeLa, a non-adrenal cell line, as previously reported, and Y1, a mouse adrenal tumor cell line [19].

In NCI-H295 cells, the analysis of apoptosis 48 h after PNU treatment showed a dose-dependent increase of apoptotic cells. These data support the proposal that inhibition of Wnt/beta-catenin signaling impairs cell proliferation mostly by increasing early and late apoptosis. In agreement, Doghman *et al*. showed that Wnt/beta-catenin inhibition by PKF115-584 repressed the entry of H295R cells into the S phase and induced their apoptosis [19]. In addition, an increase of TUNEL-positive cells, which indicates the presence of apoptotic cells, was described in *Sf1/Cre^low^*beta-catenin knockout mice, supporting the idea that the loss of beta-catenin in the adrenal cortex progressively contributes to the loss of adrenocortical tissue via apoptosis [28].

We found reduced expression of beta-catenin at the mRNA and protein level 48 h after PNU treatment. In addition, we also found a reduction of nuclear beta-catenin accumulation in PNU-treated cells, as shown by immunofluorescence. Similarly, to previous data, our results show that shorter exposure to PNU (24 h) did not affect beta-catenin expression [24]. In agreement with our results, it has been reported that PNU was able to decrease beta-catenin protein expression in juvenile zebrafish after 10 days of exposure [29]. Taken together, these data show that PNU might have a later effect and appears to require a prolonged exposure to affect gene and protein expression. Thus, PNU may directly decrease beta-catenin expression as well as its nuclear localization by directly binding to beta-catenin. Interestingly, this effect occurred 48 h after treatment with 10 and 50 μM PNU, even before the reduction of cell viability. This finding suggests that increased cell proliferation in adrenal tumor cells is driven, at least in part, by increased Wnt/beta-catenin signaling, as suggested by transgenic mouse model studies [25]. In addition, silencing beta-catenin reduced cell proliferation in H295R *in vitro* and in a xenograft mouse model [30].

We also observed that PNU led to increased mRNA expression of the beta-catenin target genes *CCND1* and *AXIN2*. In agreement, it has been shown that the Wnt/beta-catenin pathway inhibition resulted in increased *AXIN2* mRNA expression in endometrial cells [31]. Furthermore, our data suggest a potential effect on beta-catenin-dependent transcription, a mechanism of action that has been reported for other inhibitors of the Tcf/beta-catenin complex [18, 32]. The absence of an effect of PNU on *MYC* mRNA, a well-known beta-catenin target gene, supports the hypothesis that *MYC* overexpression is common in many cancers but not in ACTs [8, 33]. *TCF7* mRNA expression did not change in response to PNU treatment, supporting the hypothesis that this drug directly interferes with the Tcf/beta-catenin interaction [20].

The majority of ACCs secrete excessive amounts of cortisol, androgens or both, resulting in significant morbidity associated with excess hormone production [3]. Thus, an adjuvant therapy that, in addition to having antitumoral effects, is also able to directly decrease excessive hormone secretion is highly desirable [34]. Therefore, we also investigated the effects of inhibiting the Wnt/beta-catenin signaling on steroid production and steroidogenic enzyme gene expression. PNU treatment resulted in decreased cortisol, testosterone and, to a lesser extent, androstenedione secretion. Interestingly, adrenal steroidogenesis was impaired as early as 24 h after treatment, a time point where no decreased cell viability was yet observed. Hence, these results point to a potential direct effect of PNU on adrenal steroid secretion. This hypothesis is supported by the fact that PNU elicited reduction of corticosterone secretion but not of cell viability in Y1 cells. At higher doses it is also likely that the effect of PNU on cell decrease may contribute in part to decreased hormone production. On the other hand, we found that PNU treatment impaired the mRNA expression of *SF1* and *CYP21A2*, both important genes required for steroidogenesis, suggesting that PNU directly impairs adrenal steroid secretion by inhibiting the initial steps of steroidogenesis.

SF1 is required for StAR gene expression, which is an early and limiting step in normal steroidogenesis. Accordingly, the finding of impaired StAR protein levels after PNU treatment in NCI-H295 cells reinforces this hypothesis [35, 36]. In addition, PNU treatment also decreased the protein levels of aldosterone synthase, encoded by the *CYP11B2* gene. This result agrees with data reported by Berthon et al (2014), who showed that the inhibition of the Wnt pathway decreased hormone secretion, *CYP11B2* and *CYP21* expression after angiotensin stimulation [37]. Our findings support previous data regarding the importance of the Wnt/beta-catenin signaling in adrenal cortex homeostasis [28]. Mouse studies have suggested a critical role for beta-catenin in the maintenance of adrenal cortex progenitor cells. Partial knockout of beta-catenin in the adrenal cortex resulted in depletion of adrenocortical cells upon aging [30]. On the other hand, it has been recently suggested that the Wnt/beta-catenin signaling may control adrenal homeostasis by inhibiting fasciculata differentiation and promoting the undifferentiated state of progenitor cells, resulting in suppression of steroidogenesis [38].

There are some possible limitations to the present study. We evaluated the effects of PNU only on immortalized tumor cell lines, which are known to have possibly accumulated additional genetic defects and chromosomal instability over time. However, the NCI-H295 lineage is a good surrogate model for the analysis of the effect of the Wnt/beta-catenin signaling inhibition on ACC since it harbors the p.S45P beta-catenin mutation, as confirmed in our own stock of cells. This mutation leads to the activation of the constitutive Wnt/beta-catenin pathway [14, 15]. In addition, this lineage retains the ability to produce adrenal steroids, permitting the assessment of the effects of experimental treatments on hormonal secretion [17, 21]. One could argue that the PNU doses used were excessively high, namely the dose of 200 μM and possibly the dose of 100 μM, resulting in cytotoxic effects. However, these high doses did not result in cytotoxic effects in non-adrenal cells, such as HeLa, or in Y1 mouse adrenal cells. Furthermore, lower doses (10 and 50 μM) were able to impair H295 cell viability 96 h after treatment. These findings suggest a specific effect on cells exhibiting increased Wnt/beta-catenin signaling. In addition, we only evaluated *in vitro* effects and the results of this study do not ensure that the same effect will be obtained in *in vivo* models. The use of agents suppressing this pathway in clinical practice must be very cautious in view of toxicity and side effects since the Wnt signaling is a ubiquitous signaling pathway [32].

In conclusion, the results of this *in vitro* study demonstrated that blocking the Tcf/beta-catenin complex inhibited the Wnt/beta-catenin signaling as shown by decreased beta-catenin expression and nuclear accumulation, and impaired expression of beta-catenin target genes. These effects resulted in increased apoptosis, decreased tumor cell viability, and impairment of adrenal steroidogenesis. These promising findings may pave the way for further experiments inhibiting this pathway in pre-clinical models of ACCs. Taken together, these lines of evidence indicate that inhibitors of the Wnt/beta-catenin complex may become useful for the individualized treatment of patients with ACC presenting increased Wnt/beta-catenin signaling.

## MATERIALS AND METHODS

This study was approved by the local Ethics Committee (HCFMRP-USP #43758/2013).

### Adrenocortical tumor cell lines

The NCI-H295 and Y1 adrenocortical cell lines were kindly provided by Professor Claudimara Lotfi (University of Sao Paulo) [39]. HeLa cells were kindly provided by Dr. Paulo Peitl Jr, PhD and Dr. Beatriz Paixao, PhD (DNAapta^®^). The NCI-H295 human adrenal cell line was cultured in RPMI 1640 medium (GIBCO, Life Technologies, Foster City, CA) supplemented with 2% fetal bovine serum (Sigma Aldrich, St. Louis, MO, USA), 1% ITS Premix (BD Biosciences) and 1% penicillin/streptomycin (100 mg/mL; GIBCO Life Technologies) and harvested weekly. The Y1 mouse adrenal and HeLa human non-adrenal cell lines were cultured in DMEM medium (Sigma Aldrich) supplemented with 10% fetal bovine serum and 1% penicillin/streptomycin and harvested every 2–3 days. All cell lines were cultured in monolayer and maintained under standard conditions at 37°C with 5% CO_2_. For all experiments, cell lines were harvested at the third passage. NCI-H295 and HeLa cell lines were authenticated by analyzing the following STR markers: CSF1PO, D13S317, D16S539, D5S818, D7S820, THO1, TPOX and vWA (Supplementary Table 1).

### DNA extraction and genetic analysis of the *CTNNB1* and *TP53* genes

The analysis of exon 3 *CTNNB1* and *TP53* coding gene was performed to confirm the presence of previously described *TP53* and *CTNNB1* genetic variations in our own stock of NCI-H295 and HeLa cells. Genomic DNA was extracted from the cell lines using the QIAamp DNA Minikit (QIAGEN Inc., Valencia, CA). Exon 3 *CTNNB1* and the *TP53* coding gene were amplified by PCR and fragments were sequenced using the ABI Prism Big Dye Terminator v3.1 Cycle Sequencing Kit on an ABI 3130 instrument (Applied Biosystems, Life Technologies). PCR primers and conditions are available upon request.

### PNU-74654 (PNU)

The PNU-74654 compound (Sigma Aldrich) was resuspended in dimethyl sulfoxide (DMSO; Sigma Aldrich) at stock concentrations of 31.2 mM. For working solutions, PNU-74654 was diluted 100X in growth medium with no serum deprivation and then diluted according to the required concentrations. The half-maximal inhibitory concentration (IC_50_) of PNU-74654 for each cell line is shown in Supplementary Table 3.

### Treatment of cell lines with PNU-74654

NCI-H295 cells were plated at 200,000 cells per well in 24-well plates for gene expression, protein analysis and adrenal steroid measurements. After 48 h, cells were treated with vehicle (0.1%-0.4% DMSO) or 10, 50, 100 and 200 μM PNU-74654. After 24 and 48 h, medium supernatants were collected for adrenal steroid measurements. Adherent cells were fixed for immunofluorescence or harvested for RNA and protein isolation. At least three independent experiments were performed.

Y1 cells were plated at 200,000 cells per well in 24-well plates for gene expression and corticosterone measurement. After 24 h, cells were treated with vehicle or 10, 50, 100 and 200 μM PNU-74654. After 24 and 48 h, medium supernatants were collected for corticosterone measurements and adherent cells were harvested for RNA isolation. At least two independent experiments were performed.

HeLa cells were plated at 50,000 cells per well in 24-well plates for gene expression and protein analysis. After 24 h, cells were treated with vehicle or 50, 100 and 200 PNU-74654. After 24 and 48 h, adherent cells were harvested for RNA and protein isolation. At least two independent experiments were performed.

### Protein isolation and western blot

For protein isolation, the cells were lysed after treatment by homogenization in 100 μL IP Lysing Buffer (Pierce, Thermo Scientific) and 1 μL of Halt Protease and Phosphatase inhibitor cocktail (Thermo Scientific). Protein concentration was measured by BCA protein assay (Pierce, Thermo Scientific). Equal amounts (20 μg) of protein were subjected to SDS-PAGE, transferred to nitrocellulose membranes, blocked in TBST-T containing 5% skim milk and probed with anti-beta-catenin (dilution: 1:1000; #610154, BD Biosciences), anti-TCF (dilution: 1:1000, #sc-101170; Santa Cruz Biotechnology), anti-STAR (dilution: 1:200, #sc-25806; Santa Cruz Biotechnology) and anti-CYP11B2 (dilution: 1:500, provided by Dr. Celso Gomez-Sanchez, University of Mississipi Medical Center [40]). Anti-GAPDH (dilution: 1:1000, #sc-47724; Santa Cruz Biotechnology) was used as endogenous/loading control. Complexes were visualized with HRP-conjugated anti-mouse (dilution: 1:3000; #sc-2005; Santa Cruz Biotechnology) and anti-rabbit (dilution: 1:2000; #sc-; Santa Cruz Biotechnology) secondary antibodies and developed by enhanced chemiluminescence (Immun-Star™ WesternC™ Chemiluminescence Kit) on a ChemiDoc XRS+ System (Bio-Rad™, Hercules, CA, USA). Acquired bands were analyzed using the Image Lab™ software (Bio-Rad).

### Immunofluorescence

After treatment with PNU-74654, medium was collected and cells were fixed in methanol for 3 minutes, washed 3 times with 0.01 M PBS, and incubated with 10% normal horse serum for 1 h for blocking. Sections were then incubated overnight at room temperature with anti-beta-catenin primary antibody (dilution: 1:2000, #610154, BD Biosciences). Next, cells were washed 3 times with 0.01 M PBS, incubated with secondary antibody Cy™5-conjugated AffiniPure donkey anti-mouse antibody (Jackson Immuno Research, #715-175-150, dilution: 1:250; red color) for 4 h and washed 3 times with 0.01 M PBS. For nuclear counterstaining, cells were incubated with 4′,6-diamidino-2-phenylidone (DAPi; Cell Signaling Technology, #4083, dilution: 1:25,000) for 2 minutes, washed in 1X PBS, and slides were set with Fluoromount (Sigma-Aldrich). Expression and localization of beta-catenin were observed with a Leica TCS SP5 laser scanning confocal microscope (Leica Microsystems, Wetzlar, Germany) with fixed exposure time for all samples.

### Immunostaining

To assess the beta-catenin cellular localization in the Y1 cell line, cells were seeded over a coverslip in a 24-well plate for 24 h and fixed in methanol for 3 minutes, washed 3 times with 0.01 M PBS, and incubated with Hydrogen Peroxide Block for 10 minutes followed by Super Block (REVEAL Biotin-Free Polyvalent HRP, AMS Biotechnology, Abingdon, UK) solution for 30 minutes for blocking. Sections were then incubated overnight 4°C with anti-beta-catenin primary antibody (dilution: 1:200, #610154, BD Biosciences). Next, cells were washed 3 times with 0.01 M PBS, incubated with Complement (REVEAL Biotin-Free Polyvalent HRP, AMS Biotechnology) for 10 min and washed twice with 0.01 M PBS. Cells were then incubated with HRP conjugate (REVEAL Biotin-Free Polyvalent HRP, AMS Biotechnology) for 15 min, washed 4 times with 0.01 M PBS and incubate with peroxidase substrate chromogen (DAB; brown color). Expression and localization of beta-catenin was observed with a Zeiss microscope (Axio observer inverted microscope, Carl Zeiss, Germany).

### RNA isolation and qPCR

Total RNA from NCI-H295 cells was isolated using TRIzol^®^ according to the manufacturer's protocol (Life Technologies). RNA concentrations were quantified by spectrometry (Nanodrop 2000, Thermo Fisher Scientific Inc., Waltham, MA, USA). RNA integrity was checked according to the 28S/18S ratio, with an acceptable range of 1.6 to 2.0 and confirmed by 1.2% agarose gel electrophoresis. mRNA was reverse transcribed from 500 ng of total RNA using the High Capacity cDNA Reverse Transcription kit and MultiScribe^®^ enzyme (Life Technologies).

For quantitative Real-Time PCR (qPCR), TaqMan^®^ assays (Life Technologies, Foster City, CA, USA) were used according to the manufacturer's recommendation using cDNA (diluted 1:5) as template. TaqMan^®^ assays for NCI-H295 and HeLa cells: *CTNNB1* (Hs00170025_m1), *CCND1* (Hs00765553_m1), *AXIN2* (Hs00610344_m1), *MYC* (Hs00153408_m1), *TCF7* (Hs00175273_m1), *NR5A1/SF1* (Hs00610436_m1) and *CYP21A2* (Custom: AIVI3LV). TaqMan^®^ assay for Y1 cells: *Ctnnb1* (Mm01350391_m1). Relative expression values were determined by the 2^−ΔΔCt^ method using *GUSB* (ID: 4326320E) as endogenous control.

### Adrenal steroid measurement

Cortisol, testosterone and androstenedione concentrations were measured in the supernatant of the NCI-H295 cell medium and corticosterone concentrations were measured in the supernatant of the Y1 cell medium by radioimmunoassay (RIA) as previously described [41]. The effect of PNU effect on hormone secretion was analyzed as previously reported by Fassnacht et al. [42].

### Cell proliferation assay

The CellTiter 96^®^ AQueous One Solution Cell Proliferation Assay (Promega Corporation, Madison, WI, USA), an MTS-based assay, was used to determine cell viability before and after treatment with PNU. Briefly, cells were plated on 96-well plates at a density of 20,000 cells/well (NCI-H295) or 10,000 cells/well, incubated for 24 h (Y1 and HeLa) and 48 h (NCI-H295) and then treated with 5, 10, 50, 100 and 200 μM PNU in triplicate. After 24, 48, 72 and 96 h of incubation for NCI-H295 cells, 24 and 48 h of incubation for Y1 cells and 48 and 96 h of incubation for HeLa cells, 20 μL of CellTiter 96^®^ Aqueous Solution was added to each well and incubated for 1–3 h at 37°C with 5% CO_2_ according to the manufacturer's datasheet. Absorbance at 490 nm was obtained from a microplate reader (BioRad). At least three independent experiments were performed for each cell line.

### Stimulation of cell proliferation with forskolin

To analyze the impact of PNU under conditions of stimulation with Forskolin, NCI-H295 cells were plated on 96-well plates at a density of 20,000 cells/well, incubated for 48 h and then stimulated with 10 μM Forskolin (Sigma Aldrich) and treated with 5, 10, 50, 100 and 200 μM PNU in triplicate. After 96 h of stimulation/treatment, 20 μL of CellTiter 96^®^ Aqueous Solution was added to each well and incubated for 2 h at 37°C with 5% CO_2_ according to the manufacturer's datasheet. Absorbance at 490 nm was obtained from a microplate reader. Three independent experiments were performed.

### Detection of apoptosis with annexin V-FITC (flow cytometry)

NCI-H295 cells were plated at 350,000 cells per well in 6-well plates in triplicate. After 48 h, cells were treated with vehicle (0.08%, 0.16% and 0.32% DMSO) or with 10, 50 and 100 μM PNU. After 48 h, cells were harvested, triplicates were pooled, washed once in 0.01 M PBS and suspended in binding buffer (Sigma Aldrich) at a minimum of 1.10^6^ total cells and assayed according to manufacturer's recommendations (Annexin V-FITC Apoptosis Detection Kit, Sigma Aldrich).

The procedure basically consists of the binding of annexin V-FITC to phosphatidylserine in the cell membranes, which are beginning the apoptotic process, and the binding of propydium iodide to cellular DNA in cells where the cell membrane has been totally compromised. The cells were incubated with annexin V-FITC and propydium iodide at room temperature for 10 min and then analyzed by flow cytometry. Annexin V-FITC is detected as green fluorescence and propydium iodide as red fluorescence. Results were analyzed with the BD CellQuest Prosoftware considering 10,000 events.

### Statistical analysis

Statistical analysis was performed using GraphPad Prism 6 software (GraphPad, San Diego, CA). One-way ANOVA followed by Dunnett's multiple comparison test was used to determine differences before and after treatment with all PNU concentrations. For analysis of hormone secretion, all values are reported as mean of percentage and standard error (SEM) relative to vehicle. The level of significance was set at *p* ≤ 0.05 in all analyses. Continuous variables are reported as mean and standard error (SEM).

## SUPPLEMENTARY FIGURES AND TABLES


