# Correction: Inhibition of the Tcf/beta-catenin complex increases apoptosis and impairs adrenocortical tumor cell proliferation and adrenal steroidogenesis

**DOI:** 10.18632/oncotarget.28472

**Published:** 2023-07-07

**Authors:** Letícia F. Leal, Ana Carolina Bueno, Débora C. Gomes, Rafael Abduch, Margaret de Castro, Sonir R. Antonini

**Affiliations:** ^1^Department of Pediatrics, Ribeirao Preto Medical School, University of Sao Paulo, Ribeirao Preto, Sao Paulo, Brazil; ^2^Department of Pediatrics, School of Medicine, Federal University of Uberlandia, Uberlândia, Minas Gerais, Brazil; ^3^Department of Internal Medicine, Ribeirao Preto Medical School, University of Sao Paulo, Ribeirao Preto, Sao Paulo, Brazil

**This article has been corrected:** The Grant Support information has been updated. Please see the paragraph below:

## GRANT SUPPORT

This research was supported by the Sao Paulo Research Foundation (FAPESP; LFL fellowship 2011/10512-3; Grants 2007/58365-3 and 2011/13807-4) and the National Council of Technological and Scientific Development (CNPq; Grant 474273-2013-3). This manuscript also received funding from *CAPES* - Coordination for the Improvement of Higher Education Personnel.

Original article: Oncotarget. 2015; 6:43016–43032. 43016-43032. https://doi.org/10.18632/oncotarget.5513

