# Supplementary material for: Inhibition of the Tcf/beta-catenin complex increases apoptosis and impairs adrenocortical tumor cell proliferation and adrenal steroidogenesis
Source: Oncotarget. 2015 Oct 16;6(40):43016–32. doi: 10.18632/oncotarget.5513 (PMC4767488; doi:10.18632/oncotarget.5513)
Supplement: Supplementary file 1 [file oncotarget-06-43016-s001.pdf]

SUPPLEMENTARY FIGURES AND TABLES

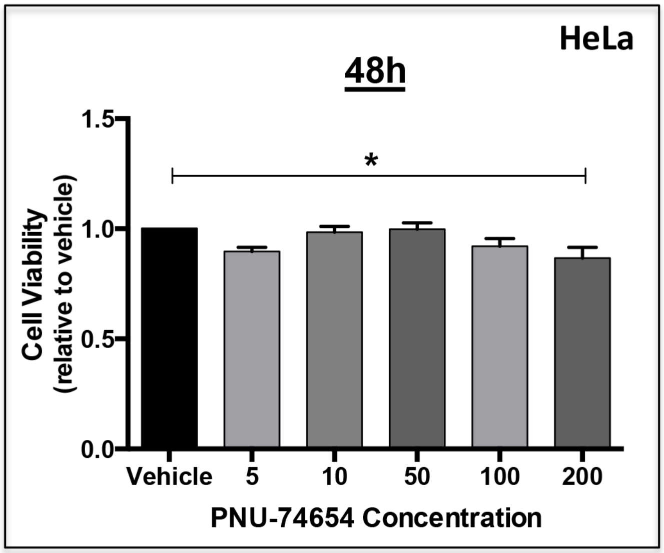

Supplementary Figure S1: PNU-74654 treatment on HeLa cell viability. No effect at 5, 10, 50, 100 µM PNU-74654. Cell viability was calculated by relative absorbance normalized by vehicle (DMSO). Values are reported as mean ± SEM. Statistics: ANOVA *$p = 0.01$.

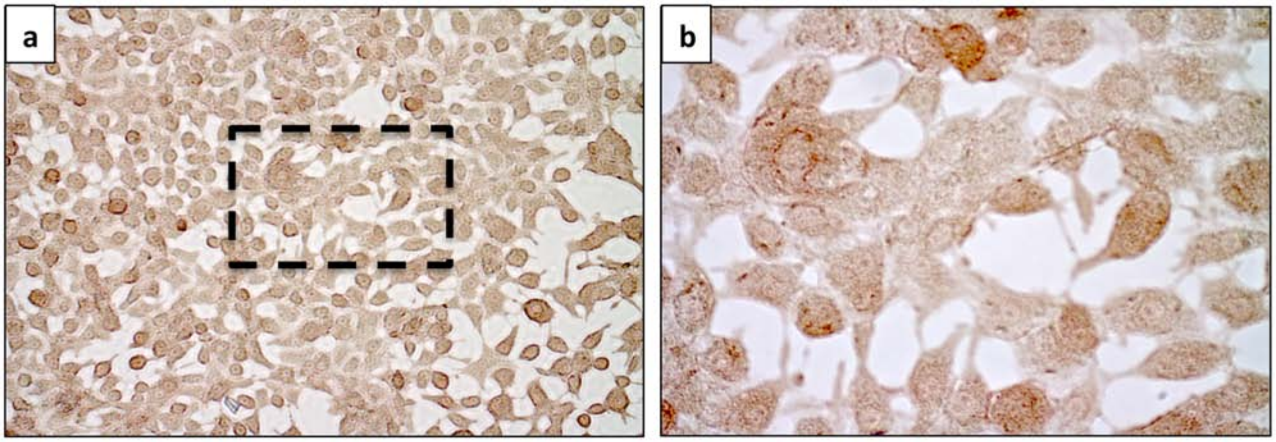

Supplementary Figure S2: Beta-catenin immunostaining in the Y1 mouse cell line. Y1 cells displayed a membranous and a weak cytoplasmic beta-catenin staining under basal conditions. Brown = beta-catenin (anti-beta-catenin antibody 1:200; BD Biosciences).

Supplementary Table S1: STR profile of human cell lines used for this study

STR	NCI-H295 (ATCC® CCL-10296)	HeLa (ATCC® CCL-2)
THO	9.3	7
TPOX	8	8/12
CSF1PO	10	9/10
D13S317	13	12/14
D16S539	11	9/10
D5S818	12	11/12
D7S820	9/12	8/12
VWA	17/18	16/18

STR, short tandem repeat; ATCC® CCL-, ATCC number.

Supplementary Table S2: Genetic variants of *CTNNB1*[1] and *TP53*[2] genes found in our own stock of NCI-H295 and HeLa cell lines

Cell lines	Exon 3 *CTNNB1*	*TP53* coding/boundary regions
NCI-H295	p.S45P	p.P278L (**COSM18654**)
HeLa	WT	p.P72R (**COSM250061**)<br>c.97–30 C > A (**g.11298C > A**)<br>c.74+38 C > G (**rs1642785**)

[1]*CTNNB1* gene: exon 3
[2]*TP53* gene: coding and boundary regions
WT: wild-type

Supplementary Table S3: IC_{50} of PNU-74654 for NCI-H295, Y1 and HeLa cell lines

Time (h)	NCI-H295	Y1	HeLa
24	129.8	563.0	648.0
48	136.6	5026	1322
72	117.2	NA	NA
96	69.34	NA	1499

Values of PNU-74654 concentration are reported as μM.
IC_{50}: half-maximal inhibitory concentration
NA: not available
h: hours
